# Low Plasma Appearance of (+)-Catechin and (−)-Catechin Compared with Epicatechin after Consumption of Beverages Prepared from Nonalkalized or Alkalized Cocoa—A Randomized, Double-Blind Trial

**DOI:** 10.3390/nu12010231

**Published:** 2020-01-16

**Authors:** Sabine Ellinger, Andreas Reusch, Lisa Henckes, Christina Ritter, Benno F. Zimmermann, Jörg Ellinger, Rudolf Galensa, Peter Stehle, Hans-Peter Helfrich

**Affiliations:** 1 Faculty of Food, Nutrition and Hospitality Sciences, Hochschule Niederrhein, University of Applied Sciences, Rheydter Str. 277, 41065 Mönchengladbach, Germany; 2 Institute of Nutritional and Food Sciences, Nutritional Physiology, University of Bonn, Nußallee 9, 53115 Bonn, Germany; areusch@uni-bonn.de (A.R.); p.stehle@uni-bonn.de (P.S.); 3 Institute of Nutritional and Food Sciences, Food Sciences, University of Bonn, Endenicher Allee 11-13, 53115 Bonn, Germany; lisa.henckes@gmx.de (L.H.); ritter.c@gmx.de (C.R.); benno.zimmermann@uni-bonn.de (B.F.Z.); galensa@uni-bonn.de (R.G.); 4 Department of Urology and Children Urology, University Hospital Bonn, Sigmund-Freud-Str. 25, 53127 Bonn, Germany; joerg.ellinger@ukbonn.de; 5 Institute of Numerical Simulation, University of Bonn, Endenicher Allee 60, 53115 Bonn, Germany; helfrich@uni-bonn.de

**Keywords:** cocoa, flavan-3-ol stereoisomers, (−)-epicatechin, (+)-catechin, (−)-catechin, plasma appearance, chiral separation, pharmacokinetics, one-compartment model

## Abstract

Flavan-3-ols are claimed to be responsible for the cardioprotective effects of cocoa. Alkalized cocoa powder (ALC), commonly used for many non-confectionary products, including beverages, provides less (+)-catechin, (−)-epicatechin, and procyanidins and more (−)-catechin than nonalkalized cocoa powder (NALC). This may affect the plasma appearance of monomeric flavan-3-ol stereoisomers after consumption of NALC vs. ALC. Within a randomized, crossover trial, 12 healthy nonsmokers ingested a milk-based cocoa beverage providing either NALC or ALC. Blood was collected before and within 6 h postconsumption. (+)-Catechin, (−)-catechin, and epicatechin were analyzed in plasma by HPLC as sum of free and glucuronidated metabolites. Pharmacokinetic parameters were obtained by a one-compartment model with nonlinear regression methods. For epicatechin in plasma, total area under the curve within 6 h postconsumption (*AUC*_0–6h_) and incremental *AUC*_0–6h_ were additionally calculated by using the linear trapezoidal method. After consumption of NALC and ALC, (+)-catechin and (−)-catechin were mostly not detectable in plasma, in contrast to epicatechin. For epicatechin, total *AUC*_0–6h_ was different between both treatments, but not incremental *AUC*_0–6h_. Most kinetic parameters were similar for both treatments, but they varied strongly between individuals. Thus, epicatechin is the main monomeric flavan-3-ol in plasma after cocoa consumption. Whether NALC should be preferred against ALC due to its higher (−)-epicatechin content remains unclear with regard to the results on incremental *AUC*_0–6h_. Future studies should investigate epicatechin metabolites in plasma for a period up to 24 h in a larger sample size, taking into account genetic polymorphisms in epicatechin metabolism and should consider all metabolites to understand inter-individual differences after cocoa intake.

## 1. Introduction

Epidemiological studies have shown that cocoa consumption lowers cardiometabolic risk [1]. This may be explained by a reduction in blood pressure [2,3,4,5,6] and serum lipids [2,7,8] and by an increase in insulin sensitivity [2,7], which could be observed in several meta-analyses of randomized controlled trials (RCTs). The beneficial effects of cocoa consumption on cardiometabolic biomarkers have been ascribed to flavan-3-ols [9,10]. In this respect, monomeric flavan-3-ols (catechin, epicatechin) are of particular interest due to their relatively high bioavailability from cocoa compared with oligomeric flavan-3-ols [11,12], which occurred in plasma only in traces (procyanidin B2) [11,12] or were even not detectable (procyanidin B5) [13].

Cocoa powder which is used for non-confectionary products, e.g., beverages, is usually alkalized to improve solubility and sensory properties. However, by alkalization, flavan-3-ols are largely destroyed, thereby reducing total flavan-3-ol content up to 80%, depending on the extent of alkalization [14,15,16]. Moreover, alkalization of cocoa powder induces an epimerization of (−)-epicatechin to (−)-catechin, an atypical stereoisomer that is also generated by roasting of cocoa beans. Consequently, alkalized cocoa powder (ALC) provides less flavan-3-ols and a lower ratio of (−)-epicatechin to (−)-catechin than nonalkalized cocoa powder (NALC) [17]. (+)-Epicatechin, also an atypical stereoisomer in processed cocoa, derives from epimerization of (+)-catechin. However, in contrast to (−)-catechin, which accounted on average for 89% of total catechin in chocolate, the amount of (+)-epicatechin was comparably low (5% of total epicatechin content) [18,19]. (+)-Epicatechin was detectable in a single alkalized cocoa powder by Ritter et al. [20], but not in the powders analyzed by Kofink et al. [17].

In rats, in situ perfusion of a solution of either pure (+)-catechin or pure (−)-catechin revealed a 3-fold lower intestinal absorption as well as 7-fold lower plasma concentrations of (−)-catechin compared with (+)-catechin metabolites [18]. In a self-experiment of a healthy volunteer of our group, the intake of a mixture of 103.5 mg (+)-catechin and 121.5 mg (−)-catechin (ratio 0.85) in milk revealed a plasma ratio of (+)-catechin to (−)-catechin metabolites to a value of 4.05. Furthermore, after consumption of a commercially available cocoa powder (46 g, dissolved in milk) providing 3.6 mg (+)-catechin and 16.6 mg (−)-catechin (ratio 0.22), the plasma ratio of (+)-catechin to (−)-catechin reached 0.8. These observations suggest a 5-fold lower bioavailability of (−)-catechin compared with (+)-catechin [20].

It is known from experimental studies that the presence of other cocoa flavan-3-ols like (−)-epicatechin or procyanidins can modulate the plasma appearance of individual flavan-3-ol stereoisomers, probably due to different affinity to metabolic enzymes or due to interactions with transport mechanisms [21,22]. Since the flavan-3-ol composition varies strongly between ALC and NALC, carefully guided human intervention studies are mandatory to monitor the plasma appearance of biologically active flavan-3-ols after consumption of cocoa products and, thus, to evaluate their potential to support cardiovascular health.

The aim of our intervention study was, thus, to follow the plasma appearance of (−)-catechin (primary aim) and of further stereoisomers, that is, (+)-catechin and (−)-epicatechin (secondary aim), after consumption of cocoa-rich beverages prepared with NALC and ALC and to compare plasma appearance of these compounds by means of pharmacokinetic parameters.

## 2. Materials and Methods

### 2.1. Study Design and Intervention

This pilot study was conducted as double-blind RCT with crossover design at the Institute of Nutritional and Food Sciences, Nutritional Physiology, University of Bonn. The study was performed according to the guidelines of the Declaration of Helsinki 2004, and the protocol was approved by the ethics committee of the University of Bonn (project-ID: 309/08). The study was registered in the German Clinical Trials Register (trial-ID: DRKS00017550) and was part of an interdisciplinary PhD program. Written informed consent was obtained from all participants.

The participants were recruited between 09.01.2010 and 08.02.2010 and were randomly assigned to groups A and B by permuted block randomization (block size of four, ratio 2:2; sequence determined by drawing lots by an uninvolved person). Both groups ingested cocoa beverages, prepared from either NALC or ALC, after at least 12 h overnight fast on two different occasions in different order. Both treatments were separated by at least one week washout. The cocoa beverages were prepared by an uninvolved person and were provided in a covered cup with a drinking straw to ensure blinding of researcher and participants. If the subjects noted that their cups were empty, a researcher removed the lids, filled the cups with a little water, and asked the participants to drink the remaining volume to ensure complete cocoa consumption. An allocation list revealing the order of both treatments (NALC, ALC) to groups A and B was sealed in an envelope, which was opened after finishing the statistical analysis.

On each study day, venous blood samples were collected before and 0.5, 1.0, 1.5, 2.0, 3.0, and 6.0 h after consumption of the cocoa beverages. After the 3 h blood sampling, the participants received a standardized meal (two rolls, each topped with 12 g butter and 40 g Gouda cheese, 48% fat in dry matter). Water was allowed to drink ad libitum.

The participants were instructed to abstain from flavonoid-rich foods (e.g., cocoa-containing foods and beverages, green tea, black tea, fruits, and juices; handout) 24 h before each treatment.

### 2.2. Participants

Twelve healthy subjects were included in the present study. They were eligible if they were between 18 and 50 years old and nonsmoker. Exclusion criteria comprised any known diseases (milk allergy, lactose intolerance, hepatic, renal and gastrointestinal diseases, metabolic and eating disorders), pregnancy, lactation, drug abuse, regular supplementation of vitamin C, vitamin E, and phytochemicals (e.g., tea or red wine extract), and participation in another trial 30 days before or throughout this study. Inclusion and exclusion criteria were checked by questionnaire.

### 2.3. Cocoa Beverages

NALC (pH 5.4–6.0) and ALC (pH 7.4–7.8) were produced following industrial standards by Schwartauer Werke, Kakao Verarbeitung Berlin, Berlin, Germany. The composition of NALC and ALC is shown in Table 1. According to manufacturer, both cocoa powders provided equal amounts of protein, fat, and carbohydrates per 100 g, and thus an equal amount of energy. As expected, the flavan-3-ol contents of ALC and NALC differed both in quantities and quality, as analyzed in our lab [20,23]. NALC contained higher amounts of flavan-3-ols compared with ALC, that is, 1.33 times more total catechin (sum of (+)-catechin and (−)-catechin), 5 times more epicatechin, and about 7 and 14 times higher amounts of procyanidin B2 and procyanidin C1, respectively. NALC provided a lower ratio of (−)-catechin to (−)-epicatechin (0.32) compared with ALC (1.26). This was expected as the ratio of (−)-catechin to (−)-epicatechin is usually <1 in nonalkalized and >1 in alkalized cocoa [24]. (+)-Epicatechin was not detectable in both cocoa powders. Hence, (−)-epicatechin was quantitatively the dominating flavan-3-ol monomer in NALC and (−)-catechin in ALC, respectively. Moreover, own analysis on methylxanthines, which was conducted according to the official methods of the German Federal Health Office [25], revealed that NALC provided more theobromine than ALC and less caffeine.

The dose of cocoa powder used for cocoa treatment was adjusted to individual body weight (BW). On the basis of our earlier pilot bioavailability studies [20], we decided to provide for both treatments equal amounts of total catechin (i.e., sum of (+)-catechin and (−)-catechin; 0.240 mg/kg BW) to allow reliable evaluation of their absorption kinetics; consequently, we used 0.6 g ALC and 0.8 g NALC per kg BW, respectively. Both cocoa powders were dissolved in heated, skimmed milk (6 mL/kg BW). All cocoa beverages were sweetened with sucrose (80 g/L) to improve taste. The total intake of energy, macronutrients, flavan-3-ols, and methylxanthines from the cocoa beverages for a subject weighing 67.7 kg is shown in Table 2.

### 2.4. Blood Sampling and Treatment of Plasma Samples

Venous blood samples were collected into tubes coated with EDTA (S-Monovette, Sarstedt, Nümbrecht, Germany) and were placed on ice immediately. Thereafter, the blood samples were centrifuged (3000× *g*, 20 min, 4 °C), and the plasma was stabilized with a solution of ascorbic acid and EDTA, as described previously [26]. All samples were stored at −80 °C for two days until (+)-catechin, (−)-catechin, and epicatechin were analyzed.

### 2.5. (+)-Catechin, (−)-Catechin, and Epicatechin in Plasma

When analyzing the concentration of monomeric flavan-3-ols in plasma, chiral separation was performed to differentiate between (+)-catechin and (−)-catechin. Since both cocoa powders provided only (−)-epicatechin and no detectable amounts of (+)-epicatechin (Table 1), only (−)-epicatechin was expected to occur in plasma after cocoa treatment. Therefore, we decided to abstain from chiral separation in case of epicatechin.

After thawing the plasma samples, each sample (500 µL) was incubated with 6250 units of β-glucuronidase type VII-A, EC 232-606-8 from *Escherichia coli* (Sigma-Aldrich, Steinheim, Germany) at 37 °C for 45 min. Thereafter, a solid-phase extraction was performed according to the procedure described previously [20]. Chiral HPLC with coulometric electrode array detection was used to determine the concentration of (+)-catechin and (−)-catechin according to the protocol of Ritter et al. [20], and HPLC with coulometric electrode array detection was also used to quantify the concentration of epicatechin according to the method of Zimmermann et al. [27]. The concentration of each flavan-3-ol in plasma was determined as sum of free, unmetabolized flavan-3-ol and glucuronidated metabolites. The limit of detection (LOD) of (+)-catechin and (−)-catechin was 23.43 nmol/L and 20.33 nmol/L, respectively. The coefficient of variation (CV) was 0.97% for (+)-catechin and 1.54% for (−)-catechin, respectively [28]. For epicatechin, the LOD was 8.27 nmol/L [27] and the CV 5.1%. For the calculation of pharmacokinetic data, concentrations below the LOD were set at the half value of corresponding detection limit to reduce bias. In addition to pharmacokinetic data which were calculated on the basis of a one-compartment model described below, the area under the curve *(AUC)* was determined for each subject as total *AUC (tAUC*_0–6h_*)* and incremental *AUC (iAUC*_0–6h_*)* by using the linear trapezoidal method based on measurements taken over 6 h.

### 2.6. Pharmacokinetic Data

The dependency of the concentration of the flavan-3-ol on time in plasma was fitted for each subject by using a one-compartment model with first-order absorption and elimination. For this, the following equation was applied [29]:

$$c\left(t\right)=\frac{D70\times k1\times k2x^{2}}{Cl\times \left(k1-k2\right)}\exp\left(-k2\times \left(t+tsh\right)-\exp\left(-k1\times \left(t+tsh\right)\right)\right),$$

where *c(t)* is the flavan-3-ol plasma concentration (µmol/L) at time *t*, *D*_70_ the dose of the flavan-3-ol that would be ingested by a person weighing 70 kg with NALC ((+)-catechin: 4 µmol ≈ 1.3 mg; (−)-catechin: 54 µmol ≈ 15.5 mg; epicatechin: 166 µmol ≈ 48.3 mg) and ALC ((+)-catechin: 2 µmol ≈ 0.6 mg; (−)-catechin: 56 µmol ≈ 16.2 mg; epicatechin: 44 µmol ≈ 12.88 mg), *k*_1_ the absorption rate (per hour), *k*_2_ the elimination rate (per hour), and *t* the time (h) recorded after complete ingestion of the cocoa drink. We included a time shift (*t_sh_*, h) in our model to consider the individual period necessary for complete ingestion of the cocoa beverages. The clearance (*Cl*) is defined as the product *V_hyp_/F_abs_* × *k*_2_, where *V_hyp_/F_abs_* denotes the hypothetical distribution volume over absolute bioavailability.

The fitting by nonlinear regression was performed with IBM SPSS for Windows, version 19.0 (IBM Corp., Armonk, NY, USA). We used the bootstrap option with 1000 bootstrap samples in order to calculate unbiased estimates of the standard deviations [30] of the parameters on plasma kinetics. We used the logarithms of *k*_1_, *k*_2_, and *Cl* as parameters to avoid negative estimates of *k*_1_, *k*_2_, and *Cl*. Further kinetic parameters were computed based on *k*_1_, *k*_2_, and *Cl*: maximum plasma concentration *C_max_* (µmol/L), the time to reach *C_max_* after cocoa intake (*t_max_*, h), absorption half-life time *t*_1/2a_ (h), elimination half-life time *t*_1/2e_ (h), *tAUC* (µmol/L × h), *V_hyp_/F_abs_* (l), and the clearance (l/h).

In order to account for the heterogeneity of the variances within the bootstrap samples for each parameter and the variances between the subjects, weighted means and variances of the parameters were determined. The weights for each parameter were chosen as τ_i_ = (σ_i_^2^ + Δ^2^)^−1^, where the within variances σ_i_^2^ were obtained by the bootstrap estimates, and the between variance Δ^2^ by the DerSimonian and Laird procedure [31], commonly used in meta-analyses.

To characterize the plasma kinetics of the flavan-3-ol stereoisomers for NALC and ALC, logarithmized parameters *k*_1_, *k*_2_, *V_hyp_/F_abs_*, *t*_1/2a_, *t*_1/2e_, *tAUC*, *C_max_*, and *Cl* were used, leading to geometric means. For *t_max_*, arithmetic means were calculated. The variation between individuals was indicated by 95% reference intervals (95% RI). To compare the plasma kinetics of the flavan-3-ols for NALC and ALC, the ratios *(k*_1_, *k*_2_, *V_hyp_/F_abs_*, *t*_1/2a_, *t*_1/2e_, *tAUC*, *C_max_)* and the difference *(t_max_)* of the parameters were calculated.

### 2.7. Anthropometric Data and Dietary Intake

On each study day, body height and weight were determined in fasting state by using a calibrated digital weighing and measuring station (seca 764, Hamburg, Germany; accuracy 0.1 kg for weight and 0.1 cm for height, respectively). On the day before each treatment, food consumption was documented by the participants in a standardized 24 h dietary record. The intake of energy, macronutrients, and dietary fiber was calculated by using the software EBISpro for Windows, version 8.0 (Erhardt, University of Hohenheim, Stuttgart, Germany), and the intake of flavonoids by using the USDA Flavonoid Database, release 3 [32].

### 2.8. Statistics

Statistical evaluation was performed by using IBM SPSS for Windows, version 19.0 (IBM Corp., Armonk, NY, USA). For details on pharmacokinetic data, see Section 2.6. Data on BW, nutritional intake before each treatment, *tAUC*_0–6h_, *iAUC*_0–6h_, and the time needed for the ingestion of each cocoa beverage were checked for normal distribution by Kolmogorov–Smirnov test. If normality could be assumed, data were compared by paired *t*-tests. Otherwise, data were log-transformed and checked again for normal distribution. If normal distribution could not be achieved, data were compared by Wilcoxon signed-rank test. Correlations between metric variables without normal distribution were investigated by Spearman’s rank correlation test. Metric data are presented as means ± standard error of the mean (SEM) if not indicated otherwise. Nominal data are given as absolute or relative frequencies.

## 3. Results

This trial was performed with 12 obviously healthy participants (6 women and 6 men; age 26.9 ± 4.1 years, mean ± SD) with a body mass index (BMI) between 18.6 and 26.4 kg/m^2^ (22.7 ± 2.6 kg/m^2^; mean ± SD). All of them completed the study. On average, both treatments were performed in intervals of 15 days. Considering a BW between 48.8 and 86.6 kg (67.5 ± 11.3 kg; mean ± SD) and a beverage consumption of 6 mL/kg BW, the volume of the ingested beverages ranged from 293 to 520 mL (405 ± 68 mL; mean ± SD). The time that was needed for complete consumption of the beverages prepared with either NALC (median 27 min, interquartile range 19–33 min) or ALC (median 34 min, interquartile range 29–42 min) was not different between both treatments (*p* = 0.168) and did not correlate with the corresponding amount of cocoa powder ingested (both *p*-values > 0.05).

Before ingestion of NALC and ALC, BW (67.7 ± 3.3 kg vs. 67.5 ± 3.3 kg) was not different, and the intake of energy (2154 ± 189 kcal vs. 2453 ± 189 kcal), protein (90 ± 7 g vs. 103 ± 11 g), fat (101 ± 11 g vs. 122 ± 16 g), carbohydrates (217 ± 27 g vs. 235 ± 28 g), dietary fiber (10 ± 2 g vs. 12 ± 2 g), and flavonoids (1.1 ± 0.6 g vs. 0.6 ± 0.2 g) on the previous day was comparable (all *p*-values > 0.05).

In fasting plasma obtained before cocoa treatment, (+)-catechin and (−)-catechin were not detectable in any sample (data not shown) and the concentration of epicatechin was below or near to the LOD in most subjects. After cocoa treatment, (+)-catechin was only detectable in 5 out of 144 plasma samples. Three of them were obtained after consumption of NALC, and two samples after ingestion of ALC. (−)-Catechin was found in 13 out of 144 plasma samples, mostly after ingestion of ALC (*n* = 10) and only in a few samples after ingestion of NALC (*n* = 3) (data not shown). Thus, pharmacokinetic parameters were not calculated for (+)-catechin and (−)-catechin.

**Figure 1:**
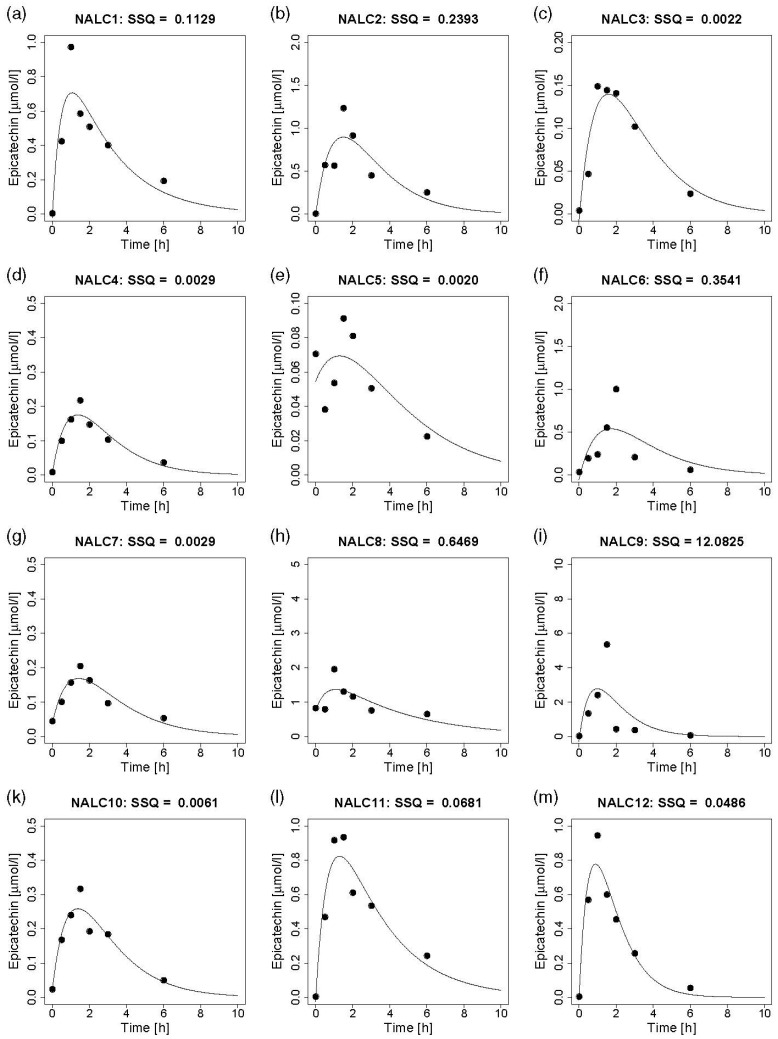
Individual epicatechin concentration time curves obtained after consumption of a beverage prepared with nonalkalized cocoa. Each panel (**a**–**m**) represents one participant. The dots represent the measured values. The curves reflect the predicted concentrations in plasma within 10 h after ingestion of nonalkalized cocoa, based on the one-compartment model. NALC, nonalkalized cocoa; SSQ, sum of squares of residuals. Note the different scaling of the y-axes.

**Figure 2:**
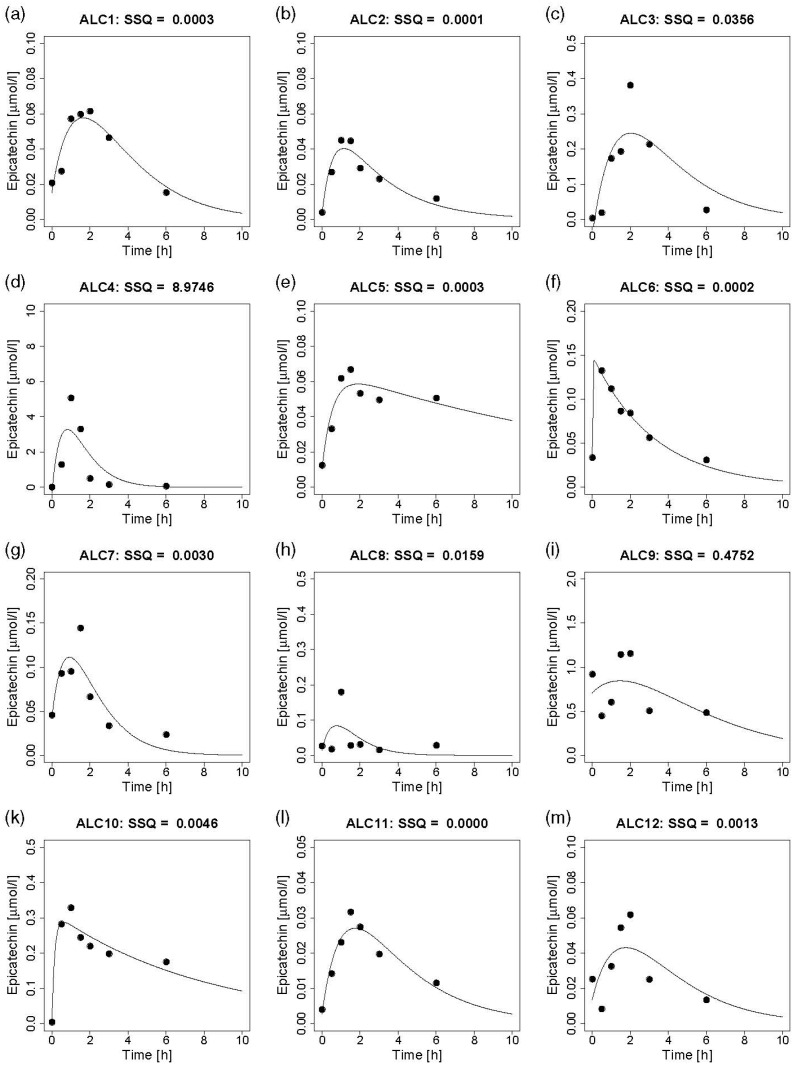
Individual epicatechin concentration time curves obtained after consumption of a beverage prepared with alkalized cocoa. Each panel (**a**–**m**) represents one participant. The dots represent the measured values. The curves reflect the predicted concentrations in plasma within 10 h after ingestion of alkalized cocoa, based on the one-compartment model. ALC, alkalized cocoa; SSQ, sum of squares of residuals. Note the different scaling of the y-axes.

In contrast to catechin enantiomers, the concentration of epicatechin was quantifiable in all plasma samples after intervention and increased in all subjects after ingestion of both NALC (Figure 1a–m) and ALC (Figure 2a–m). Each subfigure (a–m) presents the measured concentrations and the predicted concentration time curves for a single participant. For most subjects, the individual concentration time curves of epicatechin in plasma which were obtained by the model indicate a good prediction for epicatechin concentration in plasma after ingestion of NALC and ALC, respectively. In single cases, the prediction was not good, indicated by the high sum of squares of residuals (NALC9, ALC4) and the fact that the model predicts a plasma rise of only 75% (NALC1), 50% (ALC3) and <50% (ALC8, ALC12) of the maximum measured concentration. For NALC5 and ALC12, the measured concentrations do not provide a clear curve shape due to the relatively high baseline values. The increase, determined by *k*_1_, as well as the decline of the concentration–time curves, determined by *k*_2_, differed strongly between individuals. Large individual variations were also observed for *t*_1/2a,_ which depends on *k*_1_, for *t*_1/2e,_ which depends on *k*_2_, and for *t_max,_* which is determined by *k*_1_ and *k*_2_. Strong inter-individual variations also occurred for *C_max_* and for *Cl* due to the differences in *V_hyp_/F_abs_* and *k*_2_ (Table 3). The model predicts that epicatechin is almost completely eliminated from plasma by 10 h after cocoa consumption in all subjects except for one (ALC5) (Figure 1 and Figure 2).

For the comparison of both treatments (beverages prepared from NALC vs. ALC), ALC6 was excluded from our analysis as the implausible values for *k*_1_ (45.6/h; reflected by the rapid increase of the curve in Figure 2f), *t_max_* (0.10 h), and *t*_1/2a_ (0.02 h) (Table 3) would considerably confound the mean values for ALC. As shown in Table 4, mean maximum plasma epicatechin concentration after ingestion of NALC was 0.39 (95% RI: 0.24, 0.65) µmol/L (*C_max_*) and reached after 1.13 (95% RI: 0.74, 1.53) h *(t_max_)*. After ingestion of ALC, mean *C_max_* was only 0.11 (95% RI: 0.05, 0.27) µmol/L and obtained after 0.95 (95% RI: 0.36, 1.54) h (*t_max_*). As expected, the mean values of *C_max_* and *tAUC* were 3.51 (95% RI: 0.95, 12.98) and 2.82 (95% RI: 0.59, 13.55) times higher for NALC than for ALC, respectively. *K*_1_, *k*_2_, *t*_1/2a_, *V_hyp_/F_abs_*, and *Cl* were comparable between NALC and ALC, except for *t*_1/2e_ (Table 4). For *t_max_*, arithmetic means were also comparable, considering a difference between NALC and ALC values of 0.64 and the 95% RI (−0.67, 1.96).

The *tAUC*_0–6h_ for epicatechin in plasma obtained by the trapezoidal rule was higher after consumption of NALC compared with ALC (2.23 ± 0.54 vs. 1.14 ± 0.52 µmol/L × h; *p* = 0.045, based on the comparison of logarithmized values), whereas *iAUC*_0–6h_ was not different after treatment with NALC vs. ALC (1.75 ± 0.46 vs. 0.81 ± 0.45 µmol/L × h; *p* = 0.096, based on the comparison of logarithmized values).

## 4. Discussion

To the best of our knowledge, this is the first comparative human study evaluating the plasma appearance of catechin and epicatechin stereoisomers after consumption of NALC and ALC as milk drink ingredients representing common food items. Repeated plasma analyses enabled a reliable calculation of pharmacokinetic parameters based on a one-compartment model. The data confirmed our working hypothesis that (−)-catechin from cocoa powders appears only in relatively low concentrations in plasma compared with other flavan-3-ol stereoisomers if considering also the amounts ingested.

Before cocoa consumption, (+)-catechin and (−)-catechin were not detectable in fasting plasma and the concentration of epicatechin was mostly below or near to the LOD. This corresponds to our expectations with regard to the low flavonoid intake on the pre-study day and demonstrates good compliance of our participants with dietary restrictions. As BW remained stable throughout the study, changes in nutrition status which might affect pharmacokinetic parameters can be excluded.

After cocoa consumption, (+)-catechin was only detectable in 3% and (−)-catechin in 7% out of 144 plasma samples, respectively. At the first glance, this was surprising as another RCT observed an increase in catechin metabolites in plasma (no chiral separation) after ingestion of 3.05 mg catechin from cocoa up to concentrations of 0.49 µmol/L, as determined by HPLC with CoulArray detection [11]. However, in contrast to Steinberg et al. [11], sulfated metabolites were not assessed in our study, which might have underestimated the plasma appearance of both (+)-catechin and (−)-catechin.

In the meantime, the first signs on stereochemical differences in the absorption and plasma appearance of (+)-catechin and (−)-catechin, obtained from a study with rats [18] and a self-experiment of Ritter et al. [28], were confirmed by an RCT with healthy volunteers. In this study, the concentration of (−)-catechin metabolites in plasma 2 and 4 h postconsumption as well as their excretion in 24 h urine was lower after ingestion of pure (−)-catechin (1.5 mg/kg BW) compared with those metabolites obtained in plasma and urine after ingestion of equal amounts of either pure (+)-catechin or pure (−)-epicatechin, respectively. Moreover, a stereoisomeric interconversion of flavan-3-ols in vivo was not observed [33]. Thus, the lack of (−)-catechin in 90% of our plasma samples after cocoa consumption might be explained by the relatively low bioavailability of (−)-catechin, whereas the lack of (+)-catechin metabolites in more than 98% of our plasma samples may result from the comparably low intake of (+)-catechin from both NALC and ALC (Table 2).

(−)-Catechin was found in 10 plasma samples after ingestion ALC, but only in 5 samples after ingestion of NALC, despite the intake of similar amounts of (−)-catechin from NALC (0.222 mg/kg BW) and ALC (0.232 mg/kg BW; Table 2). Combined ingestion of (+)-catechin with equal doses of (−)-epicatechin (about 50 mg/kg BW) reduced bioavailability of (+)-catechin in rats [21]. If the higher intake of (−)-epicatechin relative to (−)-catechin from NALC vs. ALC (ratio 3.1 vs. 0.8) could have reduced the absorption of (−)-catechin, is questionable. For low doses, as those used in our study, absorption by a carrier-mediated transport protein as previously suggested for high doses of (+)-catechin and (−)-epicatechin in pig brush border [34] is rather unlikely. Moreover, transferability of the results from rats and *in vitro* studies to humans as well as from (+)-catechin to (−)-catechin remains questionable. If the higher intake of procyanidins from NALC might affect the absorption of catechin in our study, as suggested from a study with Caco-2 cells [22], is not clear as the procyanidins’ content was not the only difference between NALC and ALC.

Our results on plasma appearance of epicatechin after cocoa consumption are quite different to those of (+)-catechin and (−)-catechin. In contrast to catechin enantiomers, the concentration of epicatechin in all plasma samples obtained after ingestion of NALC and ALC was above the LOD, although lower amounts of (−)-epicatechin were ingested from ALC compared with (−)-catechin (Table 2). It has to be kept in mind that the LOD of epicatechin was lower (8.27 nmol/L [27]) compared with (+)-catechin (23.43 nmol/L) and (−)-catechin (20.33 nmol/L) [28]. In the study of Steinberg et al. which provided 12.2 mg monomeric flavan-3-ols from cocoa with a ratio of epicatechin to catechin of 3:1, the concentration of epicatechin metabolites in plasma was 10 times higher than those of catechin (4.11 vs. 0.4 µmol/L) [11]. Thus, our results clearly suggest that (−)-epicatechin is the major flavan-3-ol stereoisomer in plasma after cocoa treatment due to higher bioavailability compared with (−)-catechin and due to its higher abundance in cocoa than (+)-catechin.

By using a one-compartment model, absorption and excretion rates of (−)-epicatechin as well as *V_hyp_/F_abs_* could be estimated, which allows to calculate the plasma concentration time course of (−)-epicatechin and to characterize the plasma kinetics by a range of pharmacokinetic parameters. Interestingly, bioavailability of (−)-epicatechin as suggested by *C_max_* and *tAUC* obtained by the model (Table 4) roughly corresponds to the ratio of epicatechin (3.75) ingested by NALC vs. ALC. However, with regard to the *AUCs* determined by using the trapezoidal rule, the results on bioavailability are less clear as significantly higher values were only detectable for *tAUC*_0–6h_, but not for *iAUC*_0–6h_. In single cases, postprandial concentrations of (−)-epicatechin in plasma were not higher than in fasting state, which may explain the lack of significant differences in *iAUC*_0–6h_. However, inter-individual variability in epicatechin response was high, and the sample size relatively small, which makes it difficult to detect significant differences in *iAUC*_0–6h_ between both treatments. On the basis of our results, 94 subjects per group (188 in total) would be needed according to the formula of Ott [35] to detect a statistically significant difference in *iAUC*_0–6h_ of ≥0.930 ± 2.563 µmol/L × h (mean ± SD) between both treatments, presuming a power of 80%, an α of 0.05, and a β of 0.20. Hence, our study was underpowered with regard to *iAUC*_0–6h_. Shorter intervals for determining *iAUC*_0–6h_ by the trapezoidal rule would have reduced the error. When planning our study, sample size calculation was not possible as expectations on the difference (as mean ± SD) between both treatments were not available for any flavan-3-ol monomer. Nevertheless, in contrast to rats, where bioavailability of (−)-epicatechin was lower when given together with an equal dose (17.2 mmol/kg BW) of (+)-catechin [21], a lower bioavailability of (−)-epicatechin from ALC compared with NALC due to competitive effects between the absorption of catechin enantiomers and (−)-epicatechin can be nearly ruled out due to their low plasma appearance. Moreover, it is rather unlikely that the different amounts of procyanidins ingested by NALC compared with ALC (Table 2) increased the absorption of (−)-epicatechin, as suggested from a study with Caco-2 cells, as the ratio for *C_max_* (3.5) and *tAUC* (2.8) between the treatment with NALC and ALC in our model roughly corresponds to the higher intake of (−)-epicatechin intake from NALC than from ALC (Table 4). In the meantime, Caco-2 cells are no longer considered as a good model to study epicatechin absorption as the profile of (−)-epicatechin metabolites from Caco-2 cells and human enterocytes in vivo is different and determines the efflux of (−)-epicatechin metabolites back into the apical side of Caco-2 cells and to the gut lumen, respectively [36]. An RCT with healthy volunteers has recently shown that methylxanthines (1.48 mg theobromine and 0.15 mg caffeine per kg BW) enhance the absorption of (−)-epicatechin [37]. As our subjects ingested similar amounts of methylxanthines from NALC (18.55 mg theobromine, 0.58 mg caffeine) and ALC (18.42 mg theobromine, 0.88 mg caffeine) per kg BW, an impact of methylxanthines on the plasma appearance of (−)-epicatechin in our study is unlikely.

The results on *V_hyp_/F_abs_* were in the same range for NALC and ALC (Table 4). A mean value about 200 L for *V_hyp_/F_abs_* exceeds fluid compartments such as total plasma volume and total body water. After an oral ingestion of 60 mg pure [2-^14^C]-(−)-epicatechin in healthy men, only 20% of total radioactivity detected in 24 h urine was due to structurally related epicatechin metabolites (glucuronidated, sulfated, and methylated metabolites), and about half of this radioactivity refers to glucuronidated (−)-epicatechin [38]. Hence, even when sulfated and methylated metabolites were not considered, which leads to an overestimation of *V_hyp_/F_abs_*, the percentage of ingested (−)-epicatechin that reaches systemic circulation seems to be rather low.

In contrast to our hypothesis, *k*_1_, *k*_2_, *t*_1/2a_, *t_max_*, *V_hyp_/F_abs_*, and *Cl* were comparable after consumption of the beverages which had been prepared with NALC or ALC, except for *t*_1/2e_ (Table 4), suggesting that the kind of cocoa beverage which differs largely in the flavan-3-ol composition (Table 2) does not affect the velocity of absorption and metabolism of (−)-epicatechin. This is reasonable as *C_max_* was comparable after treatment with NALC and ALC, if we consider the 3.7 times larger amount of (−)-epicatechin ingested. It is important to mention that the flavan-3-ol composition was not the only factor which differed between both treatments; the intake of macronutrients was also different by using different amounts of cocoa (Table 2). In an RCT with crossover design, co-ingestion of flavanol-rich cocoa together with sugar (0.75 and 17.5 kJ/kg BW; referring to amounts about 35 and 70 g) or carbohydrate-rich foods (0.75 and 17.5 kJ/kg BW; leading to a carbohydrate intake up to 70 g) increased the *AUC* of epicatechin in plasma of healthy subjects. This effect was dependent on carbohydrate intake and could not be observed by co-ingestion of foods rich in protein or fat [39]. However, in our study, confounding effects on plasma kinetics of (−)-epicatechin by the use of different amounts of NALC and ALC are unlikely as the difference in the intake of carbohydrates between both cocoa beverages was quite low (58 g vs. 60 g) (Table 2).

The distinct kinetic curve shapes (Figure 1 and Figure 2) after both treatments reflect strong inter-individual differences in the plasma appearance of epicatechin (Table 3). Such variations were also reported to occur after ingestion of pure (−)-epicatechin [33]. Genetic polymorphisms are known to exist for sulfotransferases, but also for UDP-glucuronosyltransferases [40]. Thus, the individual variability in the extent and velocity of conjugation of (−)-epicatechin might contribute to the strong variations in absorption, metabolism, and excretion between our subjects.

A strength of our study is the chiral separation between catechin enantiomers and the use of a nonlinear model which, however, was not applicable to describe the plasma appearance of (+)-catechin and (−)-catechin due to the lack of detectable amounts of catechin metabolites in most samples. However, this model provides a good prediction on plasma kinetics of (−)-epicatechin in most subjects. Moreover, weighing individual values by considering both between and within variances has shown to be a valuable tool to compare the plasma kinetics of (−)-epicatechin after consumption of different cocoa beverages. With regard to the strong inter-individual variations and the fact that most kinetic parameters depend on each other, weighted geometric means, as calculated in our study, are more suitable than unweighted arithmetic means, which were provided in other studies investigating the plasma kinetics of (−)-epicatechin metabolites from cocoa [41,42,43].

As we did not consider sulfated flavan-3-ol metabolites, the plasma appearance of flavan-3-ol stereoisomers was underestimated, which is a clear limitation of our study. Moreover, the use of different amounts of cocoa powder to ensure an equal intake of total catechin (i.e., sum of (+)-catechin and (−)-catechin) from both treatments might introduce another confounding factor by the intake of different amounts of macronutrients. However, as stated above, this was rather unlikely in our study as the differences between both treatments were negligible (Table 2). The time required for complete ingestion of the cocoa beverages was not different between both treatments, maybe due to the strong inter-individual variability. However, the time required was considered by implication of *t_sh_* in our pharmacokinetic model. The strong inter-individual variability cannot be explained by the ingested amount of cocoa powder as it did not correlate with the time needed for complete consumption of each cocoa drink. Inter-individual differences in appetite sensations (e.g., hunger, satiety, fullness) might have affected time for ingestion of both beverages, but, of course, this remains speculative as appetite sensations were not assessed. Even if the model mostly provided good predictions, we observed for single values a discrepancy between predicted and measured concentrations in plasma (e.g., NALC1, NALC9, ALC 3, ALC8). As our model was based on seven plasma samples that were obtained within 6 h, single values with atypically high measured concentrations did not strongly affect the shape of the curve and therefore, these measured values are distant from the predicted values. The collection of blood samples was restricted to 6 h as a return to baseline value was observed in previous studies within 6 h after consumption of cocoa-containing beverages [42,44]. As the measured plasma concentrations did not return to baseline value in all participants within 6 h, we extended the predicted concentration time curves for epicatechin in plasma up to 10 h. If possible, the plasma concentrations should be determined in shorter intervals within the first 6 h and thereafter in larger intervals up to 24 h to completely assess all structurally related epicatechin metabolites [38,45].

## 5. Conclusions

In conclusion, (−)-epicatechin is the main monomeric flavan-3-ol stereoisomer in plasma after bolus ingestion of a beverage prepared from either NALC or ALC, although ALC provided lower amounts of (−)-epicatechin than of (−)-catechin. Plasma appearance of both (+)-catechin and (−)-catechin is much lower than that of epicatechin, which suggests low bioavailability of both catechin enantiomers from both NALC and ALC. Whether NALC should be preferred against ALC due to its higher (−)-epicatechin content remains unclear considering the results on *iAUC*_0–6h_. Future studies addressing similar questions should provide equal amounts of cocoa in an equal time frame to a larger sample size. They should investigate all epicatechin metabolites (i.e., glucuronidated and sulfated ones, ideally in nonmethylated and methylated form) as well as genetic polymorphisms of (−)-epicatechin metabolizing enzymes. Better predictions may be achieved by collecting more blood samples within 24 h after cocoa consumption and by considering the individual dose of epicatechin ingested in the model. The role of single factors on plasma appearance of flavan-3-ol monomers, such as the ratio of catechin to epicatechin and the impact of procyandins from cocoa, should be elucidated by matching the treatments.

## Figures and Tables

**Table 1:** Composition of the cocoa powders.

	Per 100 g Nonalkalized Cocoa Powder	Per 100 g Alkalized Cocoa Powder
Energy (kJ) ^1^	1045	1045
Macronutrients ^1^		
Protein (g)	23.5	23.5
Fat (g)	10.0	10.0
Carbohydrates (g)	14.0	14.0
Flavan-3-ols ^2^		
Catechin		
(+)-Catechin (mg)	3	1
(−)-Catechin (mg)	37	29
Epicatechin		
(+)-Epicatechin (mg)	n.d.	n.d.
(−)-Epicatechin (mg)	115	23
Oligomers (procyanidins)		
Procyanidin B2 (mg)	54	7
Procyanidin C1 (mg)	29	2
Methylxanthines ^2^		
Theobromine (mg)	3125	2326
Caffeine (mg)	97	111

^1^ According to manufacturer. ^2^ According to own analyses. Own data represent mean values, which were based on measurements in duplicate. n.d.: not detectable.

**Table 2:** Mean intake of energy, macronutrients, flavan-3-ols, and methylxanthines from beverages prepared with nonalkalized or alkalized cocoa powder in milk ^1^.

	Mean Intake from the Beverage Prepared with Nonalkalized Cocoa Powder	Mean Intake from the Beverage Prepared with Alkalized Cocoa Powder
Energy (kJ) ^2^	1398	1418
Macronutrients ^2^		
Protein (g)	22	25
Fat (g)	12	14
Carbohydrates (g)	58	60
Flavan-3-ols ^3^		
Catechin		
(+)-Catechin (mg)	1.2	0.5
(−)-Catechin (mg)	15.0	15.7
Epicatechin		
(+)-Epicatechin (mg)	n.d.	n.d.
(−)-Epicatechin (mg)	46.7	12.5
Procyanidins		
Procyanidin B2 (mg)	21.9	3.8
Procyanidin C1 (mg)	11.8	1.1
Methylxanthines ^3^		
Theobromine (mg)	1269	1261
Caffeine (mg)	39	111

^1^ Considering a mean body weight (BW) of 67.7 kg. The intake of the milk-based beverages was adapted to individual BW (6 mL/kg BW) and the use of 0.6 g nonalkalized cocoa and 0.8 g alkalized cocoa per kg BW, respectively. ^2^ Considering the intake from milk, sugar (EBISpro), and cocoa (manufacturer). ^3^ According to own analyses. Own data represent mean values, which were based on measurements in duplicate. n.d.: not detectable.

**Table 3:** Plasma kinetics of epicatechin in individual subjects after ingestion of a beverage prepared with nonalkalized and alkalized cocoa.

	*k*_1_ (h^−1^)	*k*_2_ (h^−1^)	*C_max_* (µmol/L)	*t_max_* (h)	*t*_1/2a_ (h)	*t*_1/2e_ (h)	*tAUC* (µmol/L × h)	*V_hyp_/F_abs_* (l)	*Cl* (l/h)
NALC1	1.85	0.40	0.71	1.07	0.37	1.74	2.71	154	61
NALC2	0.67	0.68	0.90	1.50	1.03	1.03	3.61	68	46
NALC3	0.64	0.64	0.14	1.60	1.08	1.08	0.59	438	280
NALC4	0.72	0.72	0.18	1.39	0.96	0.96	0.66	348	250
NALC5	0.42	0.42	0.07	1.28	1.64	1.64	0.45	880	371
NALC6	0.60	0.60	0.54	1.73	1.16	1.16	2.45	113	68
NALC7	0.81	0.49	0.17	1.44	0.86	1.41	0.74	454	224
NALC8	1.58	0.24	1.37	1.08	0.44	2.85	7.93	86	21
NALC9	1.04	1.04	2.79	1.00	0.67	0.67	7.29	22	23
NALC10	0.84	0.59	0.26	1.38	0.83	1.18	1.02	279	164
NALC11	1.40	0.38	0.82	1.29	0.50	1.82	3.52	124	47
NALC12	1.13	1.12	0.78	0.89	0.61	0.62	1.88	79	89
ALC1	0.54	0.54	0.06	1.66	1.29	1.29	0.29	283	152
ALC2	1.60	0.38	0.04	1.15	0.43	1.81	0.17	700	268
ALC3	0.52	0.52	0.25	2.04	1.32	1.32	1.27	67	35
ALC4	1.26	1.26	3.28	0.82	0.55	0.55	7.05	5	6
ALC5	1.78	0.06	0.06	1.90	0.39	11.99	1.14	673	39
ALC6	45.62	0.31	0.14	0.10	0.02	2.27	0.49	297	91
ALC7	0.90	0.90	0.11	0.93	0.77	0.77	0.34	147	132
ALC8	1.19	1.20	0.08	0.78	0.58	0.58	0.19	194	232
ALC9	0.33	0.33	0.85	1.47	2.10	2.10	6.97	19	6
ALC10	6.62	0.12	0.29	0.61	0.10	5.64	2.53	143	18
ALC11	0.83	0.34	0.03	1.75	0.84	2.03	0.15	881	300
ALC12	0.48	0.8	0.04	1.83	1.43	1.43	0.24	383	185

Data present maximum-likelihood estimates which were gained by nonlinear regression from our pharmacokinetic model. ALC: alkalized cocoa; *tAUC*: total area under the curve; *C_max_*: maximum plasma concentration; *Cl:* clearance; *k*_1_: absorption rate; *k*_2_: elimination rate; NALC: nonalkalized cocoa; *t*_1/2a_: absorption half-life time; *t*_1/2e_: elimination half-life time; *t_max_:* time to reach maximum plasma concentration; *V_hyp_/F_abs_:* hypothetical distribution volume over absolute bioavailability.

**Table 4:** Plasma kinetics of epicatechin after consumption of a beverage prepared with nonalkalized and alkalized cocoa—results from the pharmacokinetic model.

	NALC (*n* = 12)	ALC (*n* = 11)	NALC/ALC (*n* = 11)
*k*_1_ (h^−1^)	0.86 (0.66, 1.13)	0.95 (0.54, 1.68)	0.91 (0.42, 1.97)
*k*_2_ (h^−1^)	0.63 (0.49, 0.80)	0.34 (0.22, 0.52)	1.85 (1.04, 3.38)
*t*_1/2a_ (h)	0.81 (0.61, 1.05)	0.73 (0.41, 1.28)	0.76 (0.35, 1.65)
*t*_1/2e_ (h)	1.10 (0.87, 1.41)	2.04 (1.33, 3.15)	0.37 (0.21, 0.67)
*t_max_* (h)	1.13 (0.74, 1.53)	0.95 (0.36, 1.54)	n.d.
*C_max_* (µmol/L)	0.39 (0.24, 0.65)	0.11 (0.05, 0.27)	3.51 (0.95, 12.98)
*tAUC* (µmol/L × h)	1.61 (1.00, 2.58)	0.57 (0.19, 1.70)	2.82 (0.59, 13.55)
*V_hyp_/F_abs_* (l)	192 (120, 308)	211 (101, 442)	0.91 (0.32, 2.56)
*Cl* (l/h)	103 (64, 166)	78 (26, 231)	1.33 (0.28, 6.38)

Data are weighted geometric means, except for *t_max_*_,_ which is given as weighted arithmetic means and 95% reference intervals in parentheses. ALC: alkalized cocoa; *tAUC:* total area under the curve; *C_max_:* maximum plasma concentration; *Cl*: clearance; *k*_1_: absorption rate; *k*_2_: elimination rate; NALC: nonalkalized cocoa; NALC/ALC: ratio after treatment with NALC compared with treatment with ALC; n.d.: not determined; *t*_1/2a_: absorption half-life time; *t*_1/2e_: elimination half-life time; *t_max_:* time to reach maximum concentration; *V_hyp_/F_abs_:* hypothetical distribution volume over absolute bioavailability.
